# Diagnostic Performance of Indocyanine Green Plus Methylene Blue Versus Radioisotope Plus Methylene Blue Dye Method for Sentinel Lymph Node Biopsy in Node-Negative Early Breast Cancer

**DOI:** 10.1200/GO.20.00165

**Published:** 2020-08-04

**Authors:** Sanjit Kumar Agrawal, Izideen Hashlamoun, Banira Karki, Abhishek Sharma, Indu Arun, Rosina Ahmed

**Affiliations:** ^1^ Department of Breast Oncosurgery, Tata Medical Center, Kolkata, West Bengal, India; ^2^ Department of Histopathology, Tata Medical Center, Kolkata, West Bengal, India

## Abstract

**PURPOSE:**

Sentinel lymph node biopsy (SLNB) by dual-dye method (radioisotope plus blue) is the gold standard for axillary staging in patients with breast cancer, but in developing countries, logistic issues and financial constraint play a vital role. Recently, indocyanine green (ICG) has emerged as an alternative to radioisotope (technetium-99 [Tc-99]) for SLNB in breast cancer. This study compared the diagnostic performance of Tc-99 plus methylene blue (MB) dye versus ICG + MB dye SLNB.

**METHODS:**

Two hundred seven patients with early breast cancer (T1-3N0) were included in the study from 2017 to 2019. SLNB was done either with Tc-99 + MB or with ICG + MB as per availability of radioisotope. SLN identification rate (IR), SLN positivity rate, and metastatic SLN counts were compared between the 2 groups.

**RESULTS:**

IR was 199 (96%) of 207. IR was 95% in Tc-99 + MB compared with 97% with ICG + MB. The mean number of SLNs identified were 3.17 (standard deviation [SD], 1.84), with > 1 SLN identified in 87% patients by Tc-99 + MB. SLN was positive in 31.3% of patients with a metastatic SLN count of 0.37 (SD, 0.76). With ICG + MB, the number of SLNs was 2.73 (SD, 1.55), with > 1 SLN identified in 79% of patients. Twenty-eight percent of patients had positive SLNs, with a metastatic SLN count of 0.41 (SD, 0.77). A sharp decline in the availability of Tc-99 was observed, with 58% of patients in 2014 and only 12% of patients in 2018.

**CONCLUSION:**

ICG is equivalent to Tc-99 for SLNB in early breast cancer and has a good potential to be adopted by surgeons in resource-constrained setups.

## INTRODUCTION

Breast cancer is the most common malignancy in India as per GLOBOCAN 2018 estimate.^1^ Sentinel lymph node biopsy (SLNB) is the gold standard for axillary staging in node-negative early-stage breast cancer and is increasingly popular in patients with node-positive disease who were rendered node negative after neoadjuvant chemotherapy.^2^ However, in developing countries, SLNB is mostly restricted to larger centers because of technical challenges, logistics of availability and disposal of isotope, inadequate training, or financial constraints.^3^

**CONTEXT**

**Key Objective**
In developing countries, sentinel lymph node biopsy (SLNB) is not offered to all eligible patients with breast cancer because of the logistic and financial constraints of radioisotope use.
**Knowledge Generated**
Indocyanine green plus methylene blue dye SLNB has similar or superior diagnostic performance compared with radioisotope plus methylene blue.
**Relevance**
Indocyanine green dye is an alternative to radioisotope for SLNB and has good potential for adoption by surgeons in resource-constrained settings.

SLNB is best performed using a dual-dye method (radioisotope + methylene blue [MB]) with high identification rate (IR), acceptable false-negative rate (FNR), and low axillary recurrence rate.^4^ In developing countries such as India, availability of radioisotope is a major bottleneck for offering SLNB to patients with breast cancer, which has resulted in many centers either offering axillary lymph node dissection/low axillary sampling or blue dye–only SLNB.^5,6^ Published studies reported that use of blue dye–only SLNB has low IR and high FNR.^7-9^ Low axillary sampling has an acceptable FNR but is limited by excision of a higher number of lymph nodes (median, 7; range, 1-30), and long-term data on quality of life and lymphedema are still not available.^6^ In the postchemotherapy setting, use of dual-dye SLNB is a must for acceptable IR and FNR.^10^ Superparamagnetic iron oxide has recently emerged as a noninferior alternative to radioisotope for SLNB in patients with breast cancer.^11^ Its use in developing countries is limited because it is not widely available and because of high cost. Another alternative dye technique has been described in the past decade in which radioisotope is replaced by a fluorescent dye, indocyanine green (ICG).^12-14^ ICG dye is easily available and cheaper. Because it is surgeon dependent, logistics are easier with ICG than with radioisotope, which requires a nuclear medicine setup and license for radioactive substance disposal.^13^ Published studies, mainly from Japan, have shown that SLNB using ICG dye is either equivalent or better than radioisotope.^14^

In our center, there have been logistic issues related to radioisotope, which previously meant that many patients could only be offered a single-dye MB-based procedure.^15^ Since January 2017, we have used the ICG dye technique as an alternative when radioisotope SLNB was not possible, mostly because of nonavailability. SLNB was done either by isotope (technetium-99 [Tc-99]) plus MB dye or ICG + MB dye. This study compared the diagnostic performance of SLNB by Tc-99 + MB compared with ICG + MB dye in patients with node-negative early breast cancer.

## METHODS

This study is a retrospective analysis of prospectively collected data in an institutional REDCap (Vanderbilt University, Nashville, TN) database. All consecutive patients with breast cancer who had undergone upfront SLNB between 2017 and 2019 were included in the study. SLNB was done for all patients with node-negative early breast cancer (T1-3N0) per institutional protocol. Patients who had SLNB after chemotherapy were excluded from the study. All patients had dual-dye SLNB using either Tc-99 + MB or ICG + MB on the basis of availability of radioactive dye for SLNB use in the institute. Institutional review board waiver for the study was obtained (reference EC/WV/TMC/015/19).

Tata Medical Center is well equipped and authorized to perform SLNB by means of radioisotope (Tc-99m). The center has met quality indicators for SLNB per international standards, with a peer-reviewed publication in this regard.^15^

### Method of SLNB

SLNB was performed at the same time as breast surgery, with frozen section evaluation of SLNs. All patients had a dual-dye technique for SLNB using either Tc-99 + MB or ICG + MB.

#### Tc-99 + MB.

Radioactive colloid (microfiltered [0.22-μm] ^99m^Tc-S) was injected in divided doses peritumoral at least 2 hours before surgery. After induction of anesthesia, 2 mL of MB dye (1%) was injected in the periareolar region followed by 5 minutes of gentle whole-breast massage. A gamma probe (EuroProbe3, EURORAD, Eckbolsheim, France) was used to localize hot nodes. The node with maximum radioactivity and any other hot nodes that showed counts > 10% of the hottest node along with all blue-stained and suspicious nodes were removed and sent for frozen section.

#### ICG + MB.

**FIG 1:**
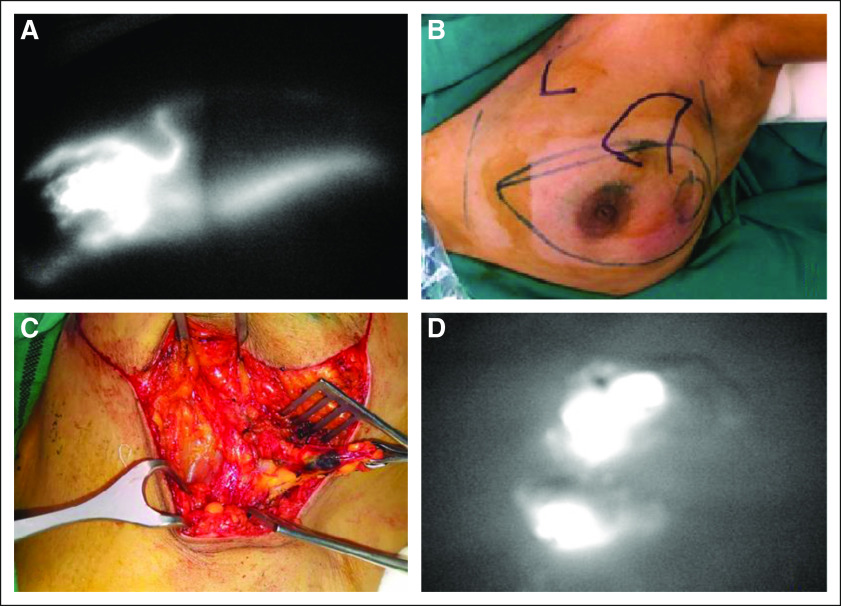
Sentinel lymph node (SLN) biopsy by indocyanine + methylene blue dye. (A) Two streams of lymphatic channels detected by fluorescence. (B) Skin marking of the lymphatic channels. (C) Skin incision followed by tracing the channel leading to a blue and fluorescent node. (D) Dissected SLN showing fluorescence.

After induction of anesthesia, 2 mL of MB dye (1%) was injected in the periareolar region, followed by 5 minutes of gentle whole-breast massage. After painting and draping, ICG (Aurogreen) 1 mL (2.5 mg) was injected subcutaneously in the periareolar area followed by 5 minutes of gentle massage. The fluorescence photodynamic eye (PDE) camera (Irillic .nm, Bangalore, India) was used to identify the fluorescent nodes. The operation theater was darkened to visualize the subcutaneous lymphatic channels with the help of a high-definition fluorescence camera system (Fig 1). Switching from normal to near-infrared light and back allowed for visualizing anatomy and fluorescence, respectively. Fluorescent lymphatic vessels were visualized and followed from the periareolar area to the axillary region to mark the incision site. The transcutaneous visibility of the lymphatic vessels was helpful to determine where exactly the skin incision should be made, and subcutaneous blue and/or fluorescent lymphatic channels were identified and followed by gentle dissection to identify the sentinel node. All fluorescent, blue, or clinically suspicious nodes were excised.

### Histopathologic Examination

Analysis of frozen section was done by a trained breast pathologist and reported in accordance with American Joint Committee on Cancer and ASCO guidelines.^16-18^ Briefly, attached fat was dissected from all the SLNs to accurately count their numbers and to measure their sizes. Lymph nodes were thinly sliced at 2-mm intervals along their longitudinal axis, and opposing surfaces were submitted to frozen section examination if the lymph node was bisected. However, if > 3 sections were present, the nonopposing surfaces of middle sections were submitted for frozen section evaluation to deter them to be > 2 mm apart. Grossly negative lymph nodes were submitted entirely for microscopy, while representative sections of grossly positive nodes were submitted for microscopy.^19^ If the initial (hematoxylin and eosin) stained section was negative for metastasis, 2-level sections were examined. The frozen section remnants were entirely submitted for permanent section evaluation. Immunohistochemistry with anticytokeratin antibody cocktail (Cytokeratin AE1/AE3; Dako, Glostrup, Denmark) was performed in tissue sections where metastatic carcinoma was suspected morphologically.

Macrometastasis was defined as a single focus of metastasis measuring > 2 mm in a given lymph node. Micrometastasis was defined as a measure > 0.2 mm but not > 2 mm. The presence of isolated tumor cell (ITC) clusters were defined as single cells or small clusters of cells not > 0.2 mm and no more than 200 cells in a single cross section.^16^

### Statistical Analysis

Data normalcy was checked by Shapiro-Wilk test. Summary statistics were expressed as percentages and means and standard deviations (SDs). Continuous variables were compared using *t* test, and categorical variables were compared using χ^2^ and Fisher exact tests as applicable. *P* < .05 was considered significant. Analysis was done using SPSS version 23 software (IBM Corporation, Chicago, IL).

## RESULTS

A total of 207 patients with node-negative early breast cancer underwent SLNB by dual dye during the study period. ICG + MB was used in 103 patients, and Tc-99 + MB dye was used in 104 patients.

Table 1 lists the patient and tumor characteristics in terms of age, body mass index, tumor size, multifocality, menopausal status, type of surgery, type of cancer with tumor grade, and hormone receptor status. The majority of patients were postmenopausal women with invasive breast carcinoma of luminal subtype. No significant statistical difference was noted in the demographic and clinicopathologic factors between the 2 groups.

**TABLE 1:**
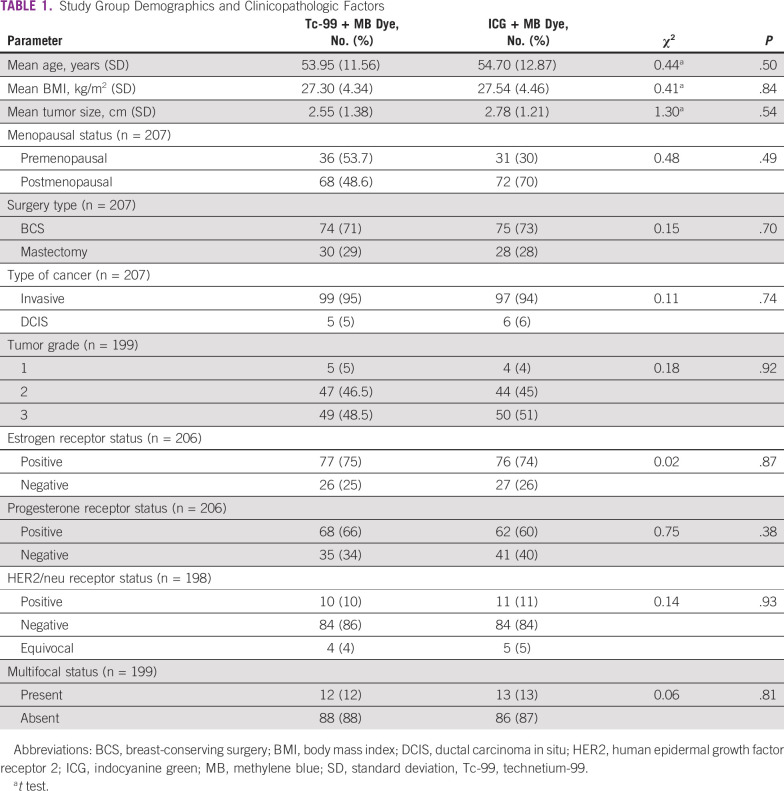
Study Group Demographics and Clinicopathologic Factors

### SLN Detection and Analysis

Of the 207 patients, SLN was identified in 199, with an IR of 96%. One hundred forty patients (70.4%) had negative SLNs, 10 (5%) had micrometastases, 47 (23.6%) had macrometastases, and 2 (1%) had ITCs (Table 2). In the 104 patients who had SLNB using Tc-99 + MB, identification of the SLN failed in 5, which resulted in an IR of 95%, whereas in the 103 patients who underwent ICG + MB SLNB, 3 showed no fluorescence or visually blue nodes, which resulted in an IR of 97%.

**TABLE 2:**
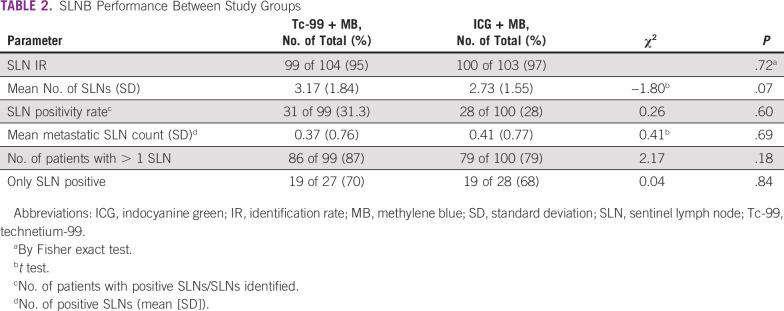
SLNB Performance Between Study Groups

In the Tc-99 + MB group, the mean number of SLNs identified was 3.17 (SD, 1.84), with > 1 SLN identified in 87% of patients, and the SLN was positive in 31 patients (31.3%), with a metastatic SLN count of 0.37 (SD, 0.76). In the ICG + MB group, the mean number of SLNs identified was 2.73 (SD, 1.55), with > 1 SLN identified in 79% of patients. Twenty-eight (28%) of 100 patients had positive SLNs, with a mean metastatic SLN count of 0.41 (SD, 0.77). On the basis of institutional protocols, all patients with macrometastatic SLN underwent axillary dissection. Two patients with micrometastases, and 2 with ITCs did not have axillary dissection. Both groups were equal in terms of the aforementioned factors, with no statistical difference (Table 2).

**FIG 2:**
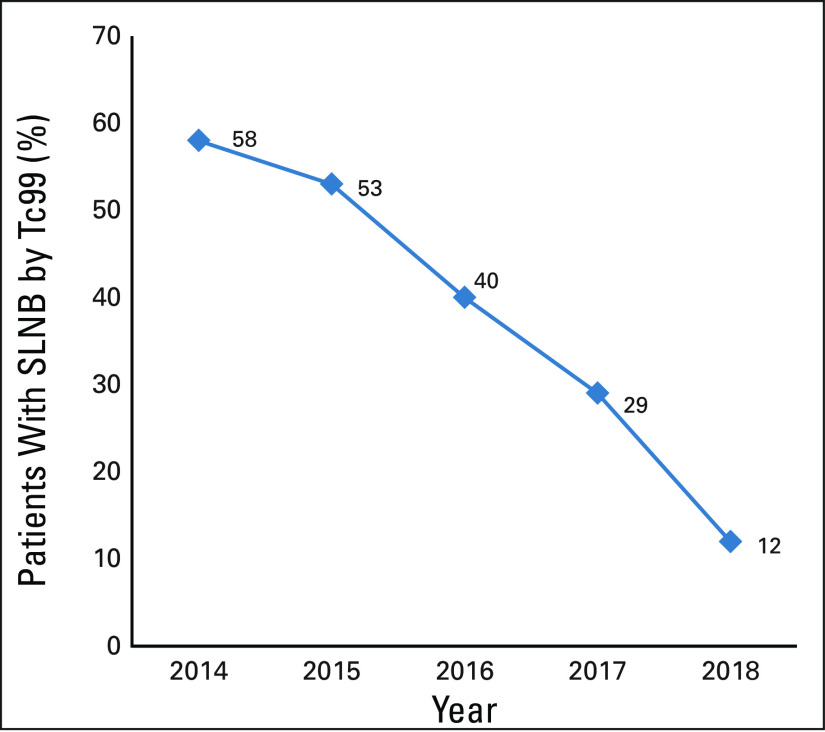
Nonavailability of radioactive dye in tertiary care cancer centers in Eastern India for sentinel lymph node biopsy (SLNB; time trend).

Figure 2 shows the nonavailability of radioactive dye in our institute. In 2014, it was available for SLNB for 58% of patients. By 2018, availability had steadily declined to 12% of patients. The reasons for this decline include government policies for radioactive dye, logistics, and financial constraints.

## DISCUSSION

This study makes a direct comparison of the diagnostic performance of ICG + MB and standard Tc-99 + MB SLNB for patients with early breast cancer. There was no statistically significant difference between the 2 groups for IR, number of SLNs removed, and metastatic SLN count, with a trend toward better axillary staging by ICG + MB. The study results confirm that the ICG + MB method may be a useful alternative to the gold standard Tc-99 + MB dye SLNB procedure for patients with breast cancer in resource-constrained developing countries.

ICG is widely available and has been used for angiography for more than a decade.^20^ It is also used for SLNB in gynecologic malignancies, in assessing vascularity of bowel resections, anastomosis, parathyroid surgery, and flap perfusion.^21-23^ Leading medical manufacturers have developed PDE, which may be combined with laparoscopic and robotic systems for ICG use in minimally invasive surgeries.

The gold standard for SLNB in breast cancer is by dual dye using radioisotope and blue dye (patent blue, isosulphan blue, MB).^4^ Radioisotope has logistic problems, like availability, legislative approval for substance disposal, nuclear medicine setup, training requirements, and cost.^24^ A systematic review reported that only 60% of eligible patients have access to SLNB worldwide because of limitations of radioactive dye use.^25^ Even in developed countries like Japan, a nationwide survey for SLNB practice showed that only 64% of eligible patients had SLNB using radioactive dye because of the aforementioned logistic problems.^26^

ICG use for SLNB has emerged as an alternative of radioisotope method of SLN identification. A meta-analysis reported a 98% detection rate for SLNB using ICG dye only, which is comparable to published results from the seminal SLNB trials using dual dye (Tc-99 + blue: NSABP32, 97.1%; ALMANAC, 96.1%).^8,9,27^ A prospective study reported a statistically significant superior IR of 99.5% with ICG + MB dye compared with either ICG alone (97%) or MB alone (89%).^28^ Another meta-analysis reported equivalent IRs for 2 single-dye techniques (ICG only, 86.9%-100%; radioisotope only, 85%-100%).^29^ Our study results show a trend toward a superior IR with ICG + MB dye (97%) compared with 95% with isotope + MB dye, although the finding was not statistically significant.

Data from a randomized controlled trial of 471 patients compared the diagnostic performance of 2 dual-dye methods (Tc-99 + MB *v* ICG + MB).^30^ To our knowledge, our study is unique in that it provides the first clinical experience to a head-to-head comparison of the diagnostic performance of 2 dual dye methods. We have listed the results of both studies in Table 3. The 2 dual-dye methods (Tc-99 + MB and ICG + MB) showed no significant difference in terms of IRs, number of SLNs removed, SLN positivity rate, and metastatic SLN count in both the trial and the clinical settings.

**TABLE 3:**
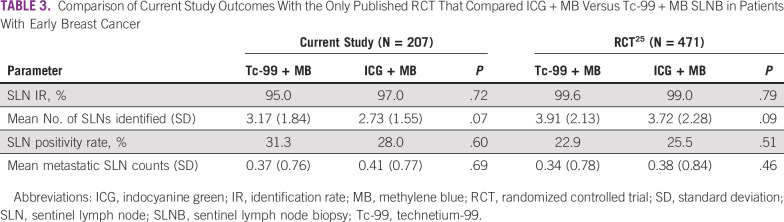
Comparison of Current Study Outcomes With the Only Published RCT That Compared ICG + MB Versus Tc-99 + MB SLNB in Patients With Early Breast Cancer

ICG is easily available and of lower cost, and its use for SLNB does not require complex facilities, like a nuclear medicine setup. The average cost for ICG + MB per patient was US $40 compared with $130 for Tc-99 + MB in our study cohort. Using a PDE camera, the surgeon can see lymphatic channels in real time in the majority of patients, which helps in incision planning in the axilla and traces the lymphatic channel just below the planned incision to the SLN. Another advantage of ICG over radioactive dye is the flexibility of case scheduling because with Tc-99, surgery needs to be done within a defined window of time (within 6 hours of dye injection), which is not always possible in a busy unit. The disadvantages reported with ICG use for SLNB are mainly a higher number of SLN removals and fluorescent contamination of the surgical field if lymphatic channels are cut accidentally while searching for SLNs.^25,30^ In our study, we found no significant difference in terms of number of SLNs removed in 2 groups (2.73 *v* 3.17; *P* = .07). To avoid leakage of ICG from the lymphatic system, which could stain the surgical field and interfere with lymph node identification, we tried for meticulous dissection and clipping of lymphatic channels before removal of SLNs. In our experience, we found it helpful to use ICG + MB, which aided with visual identification of blue-stained SLNs in an operative field with fluorescent contamination. We applied a waterproof transparent dressing just after injecting ICG to avoid spillage of dye and staining the surgeon’s. Although the dose and concentration of ICG for SLNB use is still not defined, we have used ICG in low concentration (1 mL = 2.5 mg, 1:10 dilution) to avoid fluorescence quenching, which means that a higher concentration of ICG will have a lower fluorescence. ICG in low concentration is helpful in better visualization of lymphatic channels.^31^ The main limitation of our study was that patients were not randomly assigned because our primary aim was to use the gold standard technique of Tc-99 + MB and because ICG + MB was only used in situations where we were not able to use Tc-99, although two groups of dual dye were matched for demographics and clinical factors as listed in Table 1.

In conclusion, the current study supports the use of dual-dye SLNB (ICG + MB) as an alternative method in early breast cancer, with similar or superior diagnostic performance compared with the well-established gold standard of Tc-99 + blue dye. Being nonradioactive, ICG has good potential for adoption by surgeons in resource-constrained settings. This would also support the current worldwide trend to reduce medical use of radioactive substances.
